# Implementing Thrombosis Guidelines in Cancer Patients: A Review

**DOI:** 10.5041/RMMJ.10175

**Published:** 2014-10-29

**Authors:** Dominique Farge-Bancel, Henri Bounameaux, Benjamin Brenner, Harry R. Büller, Ajay Kakkar, Ingrid Pabinger, Michael Streiff, Philippe Debourdeau

**Affiliations:** 1 Assistance Publique–Hôpitaux de Paris, Internal Medicine and Vascular Disease Unit, Saint-Louis Hospital; Sorbonne Paris Cité, Paris 7 Diderot University, Paris, France;; 2 Division of Angiology and Hemostasis, University Hospitals of Geneva and Faculty of Medicine, Geneva, Switzerland;; 3 Department of Hematology and Bone Marrow Transplantation, Rambam Health Care Campus, Haifa, Israel;; 4 Department of Vascular Medicine, Academic Medical Centre, Amsterdam, the Netherlands;; 5 Thrombosis Research Institute and Queen Mary University of London, London, UK;; 6 Division of Hematology and Hemostaseology, Department of Internal Medicine, Medical University Vienna, Vienna, Austria;; 7 Division of Hematology, Department of Medicine, Johns Hopkins University School of Medicine, Baltimore, MD, USA; 8 Institut Sainte Catherine, Avignon, France

**Keywords:** Anticoagulants, cancer, guidelines, thrombosis, venous thromboembolism

## Abstract

Venous thromboembolism is a frequent and serious complication in patients with cancer. It is an independent prognostic factor of death in cancer patients and the second leading cause of death, but physicians often underestimate its importance, as well as the need for adequate prevention and treatment. Management of venous thromboembolism in patients with cancer requires the coordinated efforts of a wide range of clinicians, highlighting the importance of a multidisciplinary approach. However, a lack of consensus among various national and international clinical practice guidelines has contributed to knowledge and practice gaps among practitioners, and inconsistent approaches to venous thromboembolism. The 2013 international guidelines for thrombosis in cancer have sought to address these gaps by critically re-evaluating the evidence coming from clinical trials and synthesizing a number of guidelines documents. An individualized approach to prophylaxis is recommended for all patients.

## BACKGROUND

Venous thromboembolism (VTE)—including deep vein thrombosis (DVT), pulmonary embolism (PE), and central venous catheter (CVC)-related thrombosis (CRT)—is a frequent and serious complication in patients with cancer.^1^ It is an independent prognostic factor of death in cancer patients and the second leading cause of death after metastasis;^2^,^3^ yet in the face of these stark facts, physicians often underestimate the increasing incidence of VTE in patients with cancer, as well as the need for adequate management.

Many factors can influence or increase the risk of VTE in a patient with cancer, including the site and type of cancer, metastasis status, type of cancer treatment (e.g. surgery, chemotherapy), use of central venous catheters, hospitalization, and patient-related factors such as age, comorbidities, and prior history of VTE.^4^–^6^ The risk of VTE also varies during the course of a malignancy, from diagnosis through treatment, remission, metastasis, and end-of-life care.^7^

Prevention and treatment of VTE in patients with cancer require the coordinated efforts of oncologists, hematologists, internists, vascular medicine specialists, and nurses, highlighting the importance of multidisciplinary management approaches aiming at personalized medicine. However, a lack of consensus among various national and international clinical practice guidelines has contributed to gaps in knowledge and practice among physicians, as well as inconsistent, inadequate prophylaxis and treatment of VTE. The 2013 international guidelines for thrombosis and cancer^2^,^8^ have sought to address these gaps, synthesizing a number of guidelines documents and recommending individualized prophylactic and therapeutic approaches for all patients.

## VENOUS THROMBOEMBOLISM IN CANCER: RISK FACTORS

There are three overarching risk factors for VTE: hypercoagulability, venous stasis, and alteration of the vascular wall, known as the Virchow triad.^9^ Specific risk factors that can induce one or more of these states are often associated with another; for example, surgery alters the vascular wall, activates coagulation, and also results in immobility that induces venous stasis.^9^ Cancer links all three conditions of the Virchow triad and puts patients with cancer at increased risk of DVT, PE, and/or CRT.^10^,^11^ Table 1 shows the patient-, cancer-, and treatment-related factors that can influence or increase the risk of VTE in a patient with cancer.^1^,^12^–^14^

**Table 1.** Risk Factors for Venous Thromboembolism.

	Risk Factors
Patient-related factors	Older age
	Obesity
	Comorbidities
	Previous VTE
	Gender
	Ethnic origin
	Thrombophilia
	Biomarkers (e.g. D-dimer, platelets, tissue factor, P-selectin, etc.)
Cancer-related factors	Cancer site, especially gastrointestinal, neurological, pulmonary, gynecological, renal, and hematological
	Histological type
	Metastatic status
	Early treatment after cancer diagnosis
Treatment-related factors	Recent surgery
	Hospitalization
	Central venous catheter
	Chemotherapy
	Hormone therapy
	Anti-angiogenic agents
	Erythropoietin

VTE, venous thromboembolism.

In a German study documenting cancer therapy and DVT risk factors of 507 patients with cancer, factors that were predictive of an increased risk of VTE included inpatient treatment (*P*<0.0001), C*-*reactive protein (*P*<0.001), chemotherapy (*P*= 0.0080), fever (*P*=0.0093), prior DVT (*P*=0.0275), and family history of DVT (*P*=0.0598).^15^ After combining factors into one variable (number of factors) the predicted VTE risk increased with the number of factors in both outpatients (odds ratio [OR] 1.85, 95% confidence interval [CI] 1.18–2.88, *P*=0.0071) and inpatients (OR 2.34, 95% CI 1.63–3.36, *P* ≤ 0.0001). When all factors were absent, the predicted risk of VTE was 2.3%; when all factors were present, predicted risk was 72%.^15^ The study also demonstrated that treatment with certain antineoplastic agents put cancer patients at a significantly higher risk of VTE, including anthracyclines (*P*=0.0466), platinum-based drugs (*P*=0.0166), and nitrogen mustard analogues (*P*=0.0411).^15^

**Figure 1.**
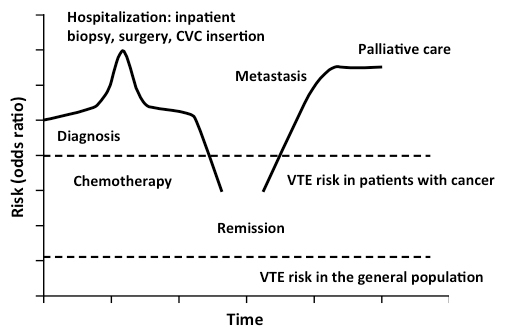
**Variation of Venous Thromboembolism Risk in a Single Patient.** CVC, central venous catheter; VTE, venous thromboembolism. Reprinted from Rao et al.^7^ with permission from Informa Healthcare.

Stage, histologic type, and grade are all important risk factors for cancer-associated VTE. There is a gradual increase of risk with the regional and distant (metastatic) stages of disease compared to the local stage (shown for lung cancer^16^ and solid tumors^17^). Chew et al. found that the hazard ratio for regional versus local disease was 1.9 (95% CI 1.6–2.2) and for distant disease, 4.0 (95% CI 3.4–4.6).^16^ Dickmann et al. found that the cumulative probability of VTE after 6 months in a cohort of 832 patients with local, regional, and distant stage cancer was 2.1%, 6.5%, and 6.0%, respectively.^17^ Interestingly, histological grade was also an important predictor for VTE. The cumulative probability of developing VTE after 6 months was higher in patients with high-grade tumors (G3 and G4, 8.2%) than in those with low-grade tumors (G1 and G2, 4.0%). Finally, the risk of VTE can vary during the course of malignancy, underlining the need for frequent patient monitoring and individualized prophylaxis and treatment (Figure 1).^7^

## VENOUS THROMBOEMBOLISM IN CANCER: EPIDEMIOLOGY

The incidence of VTE in patients with cancer has increased in recent years.^4^,^18^ This increase may be due in part to the use of newer thrombogenic chemotherapy agents as well as to the regular routine radiographic surveillance of patients with cancer and hematologic malignancies^5^ and the increased sensitivity of newer diagnostic technologies.^18^ The long-term use of CVCs facilitates chemotherapy, transfusions, parenteral nutrition, and blood samples for laboratory testing, but also increases the risk of thrombotic events.^8^,^19^

Between 4% and 20% of patients with cancer experience at least one VTE event.^20^ Approximately 20% of patients with a VTE diagnosis have active cancer,^21^ and 4% to 12% of patients with idiopathic VTE are found to have an underlying cancer.^22^ In a case–control study (1976–1990) of 625 patients with a first episode of DVT/PE age- and sex-matched to controls, cancer was shown to increase the risk of VTE 4- to 6-fold.^23^

Regardless of these findings, however, the presence and associated risks of VTE are generally underestimated in patients with cancer. In 50% of deceased patients with cancer, undiagnosed DVT or PE was found at autopsy,^22^ and in the past 10 years a significant number of unsuspected PEs have been discovered during chest scans with multi-slice computed tomography undertaken for another reason (e.g. cancer staging).

In a retrospective study of 581 patients, PE was discovered incidentally in 20 patients (3.4%), and, of these, 14 (70%) had an underlying malignancy.^24^ Similarly, in a prospective study of 385 patients with cancer, 2.6% of patients had PEs that were discovered incidentally.^25^ In a study of 135 patients with pancreatic cancer, 33% of PEs, 21% of DVTs, and 100% of visceral thromboses were discovered incidentally.^26^ In a retrospective study, cancer patients treated for incidental PE were found to have rates of recurrent VTE, bleeding complications, and mortality as high as cancer patients who developed symptomatic PE.^27^

Whether it is symptomatic or asymptomatic, however, VTE is the second leading cause of death in patients with cancer after metastasis.^1^,^28^ When cancer is diagnosed at the same time or within a year after VTE, the risk of death is three times greater at 1 year compared to patients with cancer without VTE.^23^,^29^ In hospitalized neutropenic patients with cancer, a VTE event increases the risk of death 2-fold compared to those who do not have a VTE.^30^ Leukocytosis is also strongly associated with increased risk of both VTE and mortality.^31^

Patients with cancer and a first episode of DVT are also at risk of recurrence and major bleeding complications.^32^ A recently published 35-year population-based cohort study found that survival was worse for cancer patients who had recurrent VTE and identified potential predictors of increased VTE recurrence risk, including brain, lung, and ovarian cancer; stage IV pancreatic cancer or other stage IV cancer; myeloproliferative or myelodysplastic disorders; and cancer stage progression.^33^

## VENOUS THROMBOEMBOLISM PROPHYLAXIS: MISSED OPPORTUNITIES

Venous thromboembolism is potentially preventable with the use of thromboprophylaxis in appropriate patients. For medically ill patients without contra-indications to anticoagulant therapy, several regimens have been demonstrated to be effective and well tolerated, as recommended by major guideline panels.

Nevertheless, prophylaxis continues to be under-used in both cancer and non-cancer hospitalized medical patients. The ENDORSE study, which examined practices in 358 hospitals in 32 countries, demonstrated that VTE prophylaxis as recommended by the 2004 American College of Chest Physicians (ACCP) guidelines was not implemented; it also found that a subgroup of cancer patients was insufficiently treated for VTE prophylaxis.^34^ In a retrospective cohort study of 195 patients with cancer and proven PE and in whom anticoagulant treatment was initiated, approximately half of patients received long-term treatment with vitamin K antagonists (VKAs) instead of recommended low-molecular-weight heparin (LMWH), and 20% of recurrent VTE events occurred after premature discontinuation of anticoagulant therapy.^27^

A retrospective review of electronic medication administration records over a 7-month period of adult hospitalized patients (*n*=10,516) who were ordered pharmacologic VTE prophylaxis with unfractionated heparin (UFH) or enoxaparin demonstrated that 11.9% of ordered doses were not administered. Approximately 19% of patients missed at least one-quarter of doses, and 8% of patients missed more than half of ordered doses.^35^ Patients on medical floors missed a significantly larger proportion (18%) of ordered doses compared to patients on other floors (8%, OR 2.4, *P*<0.0001); the 20% of patients who missed at least two ordered doses accounted for 80% of all missed doses.^35^

The CURVE study, a multicenter chart audit of 29 Canadian hospitals and 1,894 patients during 2003, aimed to determine the proportion of hospitalized, acutely ill medical patients who were eligible to receive thromboprophylaxis and to evaluate the frequency, determinants, and appropriateness of its use.^36^ The most frequent specified risk factor for VTE was active malignancy or cancer therapy (14% of patients), but, despite the widely known elevated risk of VTE in patients with cancer, this group was significantly less likely to receive prophylaxis than other patients (OR 0.40, 95% CI 0.24–0.68, *P*=0.0007). Non-patient factors independently associated with greater use of prophylaxis included internist versus other specialty as attending physician (OR 1.33, 95% CI 1.03–1.71) and university-associated versus community hospital (OR 1.46, 95% CI 1.03–2.07), but use of prophylaxis was unacceptably low in all groups.

In the subpopulation of the multicenter SWIVTER study patients with cancer in Switzerland,^37^ only 153 (60%) patients were receiving prophylaxis (49% pharmacological and 21% mechanical) before the onset of acute VTE.^38^ Outpatient status at the time of VTE diagnosis (OR 0.31, 95% CI 0.18–0.53), ongoing chemotherapy (OR 0.51, 95% CI 0.31–0.85), and recent chemotherapy (OR 0.53, 95% CI 0.32–0.88) were associated with the absence of VTE prophylaxis in the univariate analysis. In multivariate analysis, intensive care unit admission within 30 days (OR 7.02, 95% CI 2.38–20.64), prior DVT (OR 3.48, 95% CI 2.14–5.64), surgery within 30 days (OR 2.43, 95% CI 1.19–4.99), bed rest >3 days (OR 2.02, 95% CI 1.08–3.78), and outpatient status (OR 0.38, 95% CI 0.19–0.76) remained the only independent predictors of thromboprophylaxis. Paradoxically, although most hospitalized cancer patients were at high risk, 40% did not receive any prophylaxis before the onset of acute VTE, even in a Western country with high medical standards.

To prevent VTE and improve clinical outcomes, the Johns Hopkins Hospital implemented mandatory computerized clinical decision “smart order sets” that required physicians to assess VTE risk factors and contraindications to pharmacologic prophylaxis in hospitalized patients, including those with active cancer. Using physician responses, the order sets recommended evidence-based, risk-appropriate VTE prophylaxis. To measure the efficacy of the smart order sets, a retrospective chart review was conducted of 1,000 consecutive patients during 1 month immediately prior to (November 2007, *n*=1,000) and 1 month after (April 2010, *n*=942) the order set launch.^39^ The smart order sets significantly improved compliance with prophylaxis recommendations, resulting in significantly fewer VTE events. After implementation, risk-appropriate VTE prophylaxis increased from 65.6% to 90.1% (*P*<0.0001). Within 90 days of hospital discharge, radiographically documented symptomatic VTE decreased from 2.5% to 0.7% (*P*=0.002). Preventable harm was completely eliminated (1.1% to 0%, *P*=0.001), with no differences in major bleeding or all-cause mortality.^39^

## VENOUS THROMBOEMBOLISM TREATMENT IN PATIENTS WITH CANCER: UNDER-USE OF MANAGEMENT GUIDELINES

Guidelines on VTE treatment and prevention for patients with cancer have been successively published by the Italian Association of Medical Oncology (2006),^40^ the French National Cancer Institute (2008),^41^,^42^ the US National Comprehensive Cancer Network (2010),^43^ the European Society for Medical Oncology (2011),^44^ the ACCP (2012),^45^ and the American Society of Clinical Oncology (2013).^12^,^46^ However, low levels of guideline implementation are likely a factor in the heterogeneity of management and prophylaxis strategies found in clinical practice.^20^,^47^–^49^ Furthermore, with the exception of the French guidelines^41^,^42^ and the 2013 international guidelines,^8^ none of the above addressed the issue of CVC-related thrombosis.

### Low Levels of Guideline Implementation

There has been limited long-term use of LMWH in cancer patients to treat established VTE in preventing recurrent VTE, despite a high level of evidence-based efficacy.^50^ The CARMEN survey evaluated compliance with recommendations for the treatment of VTE in patients with cancer as detailed in the 2008 French National Institute of Cancer Standards, Options & Recommendations (SOR).^49^ The survey reviewed records of 500 patients with cancer diagnosed with a VTE in 47 French centers and found that while most patients received adequate initial VTE treatment for the first 10 days, only 60% were treated according to guideline recommendations after 10 days and in the face of hematologic malignancy (Table 2). Two other recent studies had similar findings.^51^,^52^

**Table 2.** Carmen Survey—Venous Thromboembolism Treatment.

Treatment	Patients (%) Who Received VTE Treatment^*^
Initial treatment (0–10 days)	98%
Treatment after 10 days (without severe renal insufficiency)	62%
Treatment after 10 days (with severe renal insufficiency)	25%
Treatment of VTE in cases of hematologic malignancy	20%

* As recommended by the 2008 French National Cancer Institute (data from Farge et al.^42^).

VTE, venous thromboembolism treatment.

In a small international study of 141 thrombosis (*n*=65, 46%) and non-thrombosis (*n*=76, 54%) specialists, LMWH was selected as the first choice for initial (5–10 days) and long-term treatment (3– 12 months after initial treatment period) of VTE by 83% (116/141) of respondents, but only 60% (70/116) of those prescribed full therapeutic doses throughout the treatment period; the balance (40%) chose a dose reduction.^53^ While this survey demonstrated a relatively high use of LMWH for the long-term treatment of VTE in patients with cancers, only 32% (37/116) of respondents indicated that they would continue anticoagulants in all patients after 3–12 months; another 49% (57/116) said they would continue anticoagulant therapy in most patients, 17% (20/116) in some patients, and 2% (2/116) would not continue treatment in any patient.^53^ Of those respondents continuing an anticoagulant after 3–12 months, the chosen type of anticoagulant varied widely: 44% selected LMWH, 10% selected a VKA, and 45% selected either LMWH or VKA per individual patient.^53^ There was also disparity in treatment strategies for recurrent VTE.^53^ For patients on LMWH therapy at the time of recurrence, 42% were prescribed LMWH at a higher dose, 8.2% were switched to a combination of LMWH and VKA, 4.3% were switched to a VKA, 30% were switched to a vena cava filter with or without an anticoagulant, and 15% were referred to a vascular/hematology specialist. For patients on VKA therapy at the time of recurrence, 84% were switched to a LMWH, 8.6% were switched to a vena cava filter, and 3.6% were switched to a combination of LMWH and VKA.

### Perceived High Cost

Treatment of VTE in patients with cancer also poses a significant burden for the health care system in terms of resource utilization. A Canadian analysis of data from the CLOT trial database measured the economic value of the LMWH dalteparin versus warfarin for recurrent VTE prophylaxis.^54^ While patients in the dalteparin group had significantly higher overall costs versus the warfarin group (C$4,162/patient versus C$2,003, *P*<0.001), dalteparin proved to be a cost-effective intervention for patients with cancer at risk of recurrent VTE when other factors were taken into account, including efficacy, need for patient monitoring, clinical burden, patient preference, and quality-adjusted life years (QALYs). Importantly, the costs associated with dalteparin prophylaxis fell well below the C$50,000 cost per QALY threshold used by Canadian health care systems as the acceptable economic value when considering new medical interventions.^54^

Similarly, a US study of health care costs associated with VTE in ambulatory cancer patients found that VTE was associated with significant resource utilization (both inpatient and outpatient), but that measures to prevent VTE may reduce health care utilization and costs.^55^

### Perceived Intolerance Related to the Long-Term Use of LMWH

Clinicians may avoid the long-term use of LMWH in patients with cancer due to presumed intolerance and low patient acceptance of subcutaneous injections.^56^ Many oncologists do not prescribe LMWH for 3 or 6 months to avoid additional treatment burden for patients,^20^ but such concerns may not be valid (Table 3).^56^ It has been demonstrated that, similar to patients with diabetes, cancer patients can cope with the injections as long as they understand their benefits.^57^

**Table 3.** Adverse Effects of Long-Term Use of Low-Molecular Weight Heparin and Vitamin K Agonist.^*^

**Study**	***n***	**Treatment**	**Thrombocytopenia (%)**	**HIT (%)**	**Bruising (%)**	**Allergy (%)**	**Fracture (%)**
CANTHANOX	71	VKA	24	0	–	–	0
	67	LMWH	32	0	–	–	0
CLOT	336	VKA	–	–	–	–	–
	336	LMWH	–	–	–	–	–
LITE	100	VKA	4	–	–	–	5
	100	LMWH	6	–	–	–	3
ONCENOX	34	VKA	14	0	–	–	–
	68	LMWH	3	0	–	–	–
MALT	154	Placebo	–	0	–	1	–
	148	LMWH	–	0	–	0	–
FAMOUS	184	Placebo	–	–	–	–	–
	190	LMWH	–	–	–	–	–
SIDERAS	70	Placebo	2	–	19	–	–
	68	LMWH	5	–	50	–	–

* *n*=500 patients with cancer. HIT, heparin-induced thrombocytopenia; LMWH, low-molecular weight heparin; VKA, vitamin K agonist. Reprinted with permission from Debourdeau et al.^56^

Thrombocytopenia is generally associated with simultaneous use of chemotherapy and bone marrow infiltration, rather than with the use of heparin.^56^ In fact, thrombocytopenia related to heparin use in patients with cancer is more frequent with UFH than LMWH^56^ and, in most cases, is related to cancer treatment. The risk of bone fractures is also not increased with LMWH.^20^ Two studies in palliative care have demonstrated that LMWH treatment was well accepted and had a positive impact on quality of life.^58^,^59^

### Guideline-Related Issues

Although there are several national and international guidelines for the prevention of VTE and treatment of established VTE in patients with cancer, including CRT, a number of issues may affect the quality and therefore the acceptance of the guidelines, including scope and purpose, methodological approach, quality of methodology and validation, and lack of consensus on prophylaxis and treatment between guidelines.^42^,^46^–^48^ For this reason, the 2013 international guidelines were developed using a panel of international experts: to homogenize practice and be applicable at all regional and national levels (Table 4).

**Table 4.** Venous Thromboembolism Guidelines.

	**AIOM^40^**	**SOR^41^,^42^**	**NCCN^43^**	**ACCP^45^**	**ASCO^46^**	**International Guidelines^2^,^8^**
DVT/PE prophylaxis	Yes	No	Yes	Yes	Yes	Yes
DVT/PE treatment	Yes	Yes	Yes	Yes	Yes	Yes
CRT prophylaxis	Yes	Yes	No	Yes	No	Yes
CRT treatment	No	Yes	Yes	Yes	No	Yes
Methods of CVC insertion	No	Yes	No	No	No	Yes
Target patient population	Yes	Yes	Yes	Yes	Yes	Yes
Grading of recommendations	Yes	Yes	Yes	Yes	No	Yes
External reviewers	Yes	Yes	No	Yes	No	Yes

ACCP, American College of Chest Physicians; AIOM, Italian Association of Medical Oncology; ASCO, American Society of Clinical Oncology; CRT, catheter-related thrombosis; CVC, central venous catheter; DVT, deep vein thrombosis; NCCN, National Comprehensive Cancer Network; SOR, Standards, Options & Recommendations (French national guidelines); VTE, venous thromboembolism.

## 2013 INTERNATIONAL GUIDELINES

In 2013, an international group of experts was established to synthesize the different existing guidelines, enhance implementation,^1^ and address the need for international guidelines that could be applied in regions outside of those that had already developed their own guidelines.^2^ These guidelines—on the prophylaxis and treatment of established VTE in patients with cancer^2^ and the prophylaxis and treatment of established thrombosis associated with central venous catheters in patients with cancer^8^—address important clinical issues, including the following:

- Initial treatment of diagnosed VTE (0–10 days)
- Maintenance treatment (10 days–3 months) and long-term treatment (>3 months) of diagnosed VTE
- Treatment of recurrent VTE with VKA, LMWH, and vena cava filter
- VTE prophylaxis in cancer surgery
- Prophylaxis in medical cancer patients, with special focus on lung, pancreatic, and myeloma patients as well as appropriate use of devices
- Treatment of established CRT
- CRT prophylaxis
- Specific patient populations: brain tumors, neurosurgery, renal failure, thrombocytopenia, pregnant women with cancer

For each of the above issues, the results of a literature search (published articles between January 1996 and January 2011, prospectively continued up to June 2011) and analysis were summarized and discussed by a working group of 24 experts from various specialties (oncology, hematology, internal medicine, vascular medicine, biology, and epidemiology). Overall conclusions with corresponding levels of evidence were formulated on the basis of pooled results and conclusions for each issue and the degree of agreement between the studies, using the Grading of Recommendations Assessment Development and Evaluation system. The guidelines were then peer-reviewed in February 2012 by 42 independent experts worldwide, encompassing all medical and surgical specialties involved in the management of patients with cancer, and by three volunteer patient representatives.

### Treatment and Management of Established VTE in Patients with Cancer

For initial treatment of established VTE in patients with cancer, LMWH is recommended, although fondaparinux and UFH can be also used. Vena cava filters may be considered, and thrombolysis may be considered only on a case-by-case basis.^2^

For early maintenance and long-term treatment, LMWH for a minimum of 3 months is preferred over VKAs. After 3 to 6 months, LMWH or VKA continuation should be based on individual evaluation of the benefit–risk ratio, tolerability, patient preference, and cancer activity.^2^

For treatment of VTE recurrence under anticoagulation, three options are recommended: a switch from VKA to LMWH when treated with VKA; an increase in LMWH dose when treated with LMWH; and, in some patients, vena cava filter insertion.^2^

### Prevention of VTE in Patients with Cancer

In surgical cancer patients, the use of LMWH or low doses of UFH is recommended; it should be started 12 to 2 hours preoperatively and continued for at least 7 to 10 days.^2^ Extended prophylaxis (4 weeks) after major laparotomy may be indicated in patients with cancer who have a high risk of VTE and low risk of bleeding. Mechanical methods are not recommended as monotherapy except when pharmacological methods are contraindicated.

In medical cancer patients, prophylaxis with LMWH, UFH, or fondaparinux is recommended.^2^ For patients with acute lymphocytic leukemia treated with L-asparaginase, prophylaxis may be considered in some patients, but, in other patients receiving chemotherapy, prophylaxis is not routinely recommended. However, in patients treated with thalidomide or lenalidomide combined with steroids and/or chemotherapy, VTE prophylaxis is recommended.

### Treatment and Prevention of Thrombosis Associated with CVC in Patients with Cancer

In symptomatic CRT, anticoagulant treatment is recommended for a minimum of 3 months.^8^ In this setting, LMWHs are suggested; VKAs can also be used in the absence of direct comparisons of these two types of anticoagulants. The CVC can be kept in place if it is functional, well positioned and non-infected, and there is good resolution under close surveillance. However, whether the CVC is kept or removed, no standard approach in terms of anticoagulant treatment duration has been established.

In patients with CVC, use of anticoagulant treatment for routine prophylaxis of CRT is not recommended.^8^ Central venous catheters should be inserted on the right side, in the jugular vein, and the distal extremity of the CVC should be located at the junction of the superior vena cava and the right atrium.

## FUTURE CONSIDERATIONS FOR GUIDELINES

Future iterations of comprehensive clinical guidelines should address the role of new oral anticoagulant agents for the treatment of VTE in cancer patients, as well as other specific clinical scenarios, such as prevention of VTE during transient periods of immobility at home, and treatment of incidental VTE.

### New Oral Anticoagulants

The experts of the international working group acknowledged the potential benefit of new oral anticoagulant agents for the treatment of VTE in cancer patients.^2^,^8^ At the time of publication in 2013, the group considered it was premature to issue recommendations or guidance on the use of these new agents in this setting in view of the absence of specific data, and considering that none of these products had yet been approved for use for VTE treatment and none of the experts had enough clinical experience with their use to give any meaningful “best practice advice.”

### Prevention of VTE during Transient Periods of Immobility

As thromboembolism is a leading cause of death in cancer patients receiving outpatient chemotherapy,^60^ future guidelines should address specific prophylaxis recommendations for ambulatory patients who are temporarily immobile due to adverse effects of cancer treatments. According to the 2013 international guidelines, prophylaxis is not recommended routinely for patients receiving chemotherapy, though primary pharmacological prophylaxis of VTE may be indicated in patients with locally advanced or metastatic lung cancer treated with chemotherapy and having a low bleeding risk.^2^

### Treatment of Incidental VTE in Patients with Cancer

Evidence supporting the use of anticoagulation in patients with incidental DVT or PE is scant. It has been suggested that anticoagulant therapy is warranted if there are no contraindications.^61^ In cases where the diagnosis is questionable, appropriate testing (e.g. CT pulmonary angiogram or compression venous ultrasonography) should be undertaken to confirm the diagnosis before treatment is initiated.^61^

## CONCLUSION

Cancer-associated VTE poses a significant threat to patient prognosis and a major challenge for clinicians treating complex patients. This complication among patients with cancer is serious, and its frequency has been increasing in recent years. With the recent publication of 2013 international guidelines for the appropriate prophylaxis and treatment of VTE, including CRT, there is a significant opportunity not only to educate health professionals about the severity of this common complication, but also to disseminate these new recommendations and facilitate their implementation globally using all the tools at our disposal, including mobile and online technology.

## Abbreviations:

ACCP: American College of Chest Physicians;
AIOM: Italian Association of Medical Oncology;
ASCO: American Society of Clinical Oncology;
CI: confidence interval;
CRT: catheter-related thrombosis;
CVC: central venous catheter;
DVT: deep vein thrombosis;
HIT: heparin-induced thrombocytopenia;
INCa: French National Institute of Cancer;
LMWH: low-molecular-weight heparin;
NCCN: National Comprehensive Cancer Network;
OR: odds ratio;
PE: pulmonary embolism;
QALY: quality-adjusted life-year;
SOR: Standards, Options & Recommendations
UFH: unfractionated heparin;
VKA: vitamin K agonists;
VTE: venous thromboembolism.
